# Case report: Primary cardiac synovial sarcoma with suspected connective tissue disease diagnosed by EBUS-TBMB

**DOI:** 10.3389/fmed.2025.1515233

**Published:** 2025-02-05

**Authors:** Yanmei Feng, Chunxia Wu, Jing Chi, Linying Li, Pu Wang, Rui Guo

**Affiliations:** ^1^Department of Respiratory Medicine, Chongqing Emergency Medical Center, Chongqing University Central Hospital, Chongqing, China; ^2^Department of Respiratory and Critical Care Medicine, The First Affiliated Hospital of Chongqing Medical University, Chongqing, China; ^3^Center for Excellence in Brain Science and Intelligence Technology (CEBSIT), Institute of Neuroscience(ION), Chinese Academy of Sciences, Beijing, China; ^4^Department of Emergency Medicine, The First Affiliated Hospital of Chongqing Medical University, Chongqing, China

**Keywords:** primary cardiac synovial sarcoma, antinuclear antibodies, pleural effusion, connective tissue disease, EBUS-TBMB

## Abstract

Primary cardiac synovial sarcoma (PCSS) most commonly originates in the right atrium of the heart and is exceptionally rare. Although biomarkers of autoimmune diseases, such as antinuclear antibodies (ANAs), have been reported as potential indicators of certain tumors, the association between PCSS and ANAs remains unclear. Herein, we describe a case of pleural effusion that was initially considered to be due to connective tissue disease (CTD) but was finally diagnosed as PCSS through endobronchial ultrasound-guided transbronchial mediastinum biopsy (EBUS-TBMB). Clinicians need to update their knowledge regarding the potential association between PCSS and ANAs. This case report also emphasizes the importance of EBUS-TBMB, under the guidance of positron emission tomography/computed tomography (PET/CT), in the diagnosis of this rare tumor in an unusual location.

## Introduction

Primary cardiac synovial sarcoma (PCSS) is an exceptionally rare entity, accounting for approximately 5% of cardiac sarcomas and less than 1% of all primary cardiac tumors (1). The left atrium is the most common site for this condition, with the majority of cases originating in the upper part of the pericardium. The symptoms of PCSS are diverse; however, the most prevalent manifestations among male individuals are dyspnea and cough (2). The diagnosis of synovial sarcoma (SS) relies on a biopsy in combination with several findings such as characteristic morphology, immunohistochemical profile, and the identification of the driver translocation. Transducing-Like Enhancer (TLE1) has been proven to be a sensitive and specific marker for SS (3). Cluster of differentiation 99 (CD99) was also detected in 60% of SS cases (4). Anti-nuclear antibodies (ANAs), which are crucial for diagnosing autoimmune diseases, have emerged as mysterious elements in the complex field of tumorigenesis. Studies have shown that ANAs could potentially serve as early markers or indicators of certain malignancies, such as lung and colon cancers (5, 6). Initial surgical resection biopsy is the primary method for obtaining tissue samples of PCSS (7, 8). However, it is considerably more difficult and hazardous to perform a surgical biopsy due to the tumor’s unique location and propensity for early metastasis. Moreover, the association between PCSS and ANAs has not been reported.

Herein, to improve clinicians’ comprehension of atypical PCSS and the novel biopsy approach, we present a case of a female patient who presented with a mass in the vena cava and the right atrium, along with pleural effusion and elevated ANA titers. Eventually, the patient was diagnosed with PCSS through a safer biopsy method, namely endobronchial ultrasound-guided transbronchial mediastinum biopsy (EBUS-TBMB) under the guidance of positron emission tomography/computed tomography (PET/CT).

## Case presentation

**Figure 1:**
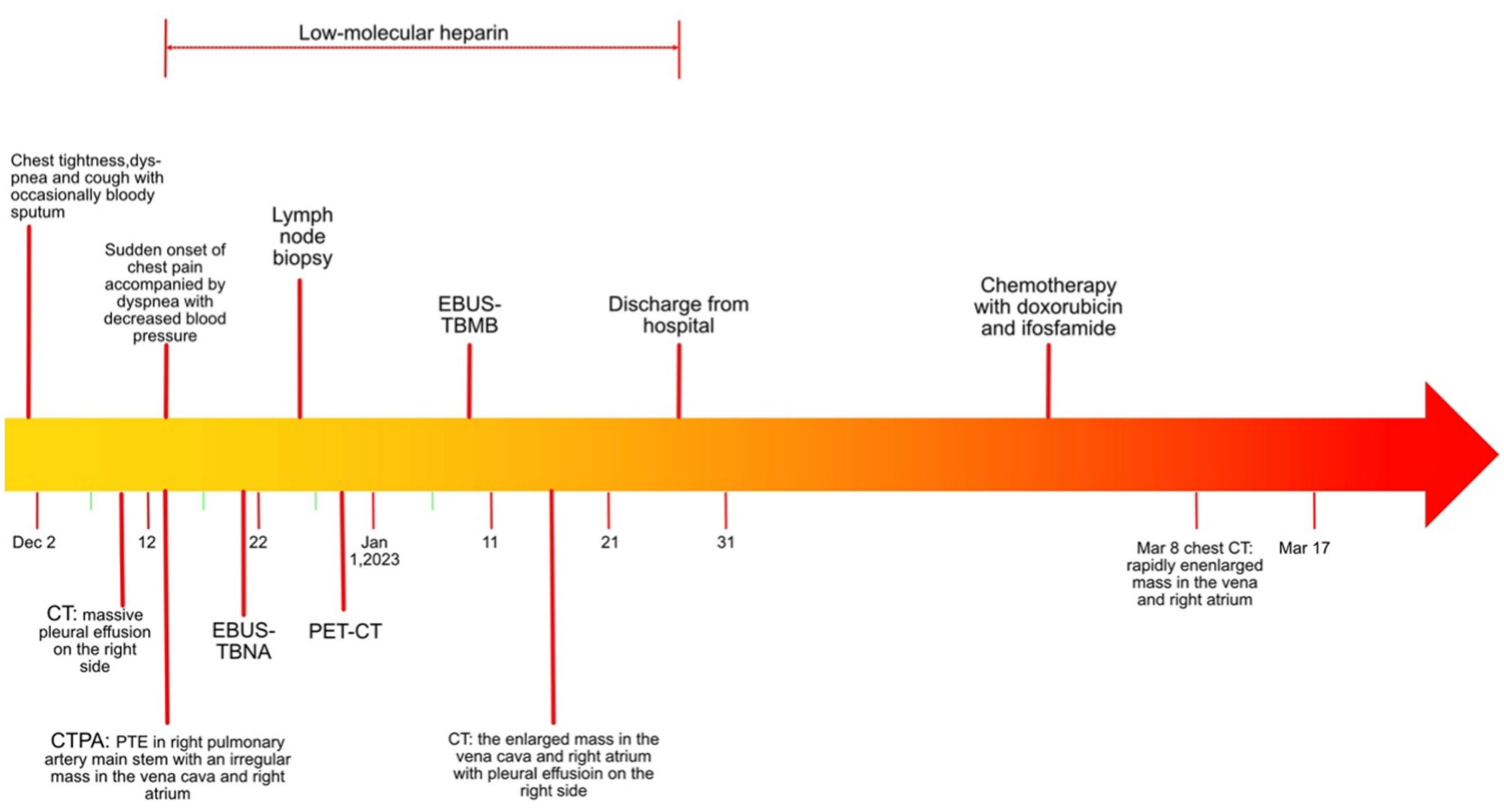
Timeline of the clinical course and treatment.

A timeline of the clinical course and treatment is shown in Figure 1. A 53-year-old woman was admitted to our hospital on 9 December 2020 due to chest tightness and dyspnea for 1 week, accompanied by cough with occasional bloody sputum. There was no history of hemoptysis, recent exposure to tuberculosis or irritant gases, or any other underlying health conditions, except for a family history of lung cancer in her father. An unenhanced chest computed tomography (CT) was performed at a local hospital, revealing a massive right pleural effusion (data not shown). Physical examination indicated normal vital signs (body temperature: 36.2°C, pulse rate: 98/min, respiratory rate: 20/min, and blood pressure: 109/86 mmHg); however, asymmetric breath sounds were noted, without the presence of rales or wheezing. Neck thickness and edema in both upper limbs were detected. Blood gas analysis revealed a pH of 7.46, a partial pressure of carbon dioxide (PCO_2_) of 37 mmHg, an oxygen partial pressure (PO_2_) of 74 mmHg, a bicarbonate (HCO3^−^) level of 27.8 mmol/L, and an oxygen saturation (SO_2_) of 96%. Additionally, her leukocyte count was normal.

**Figure 2:**
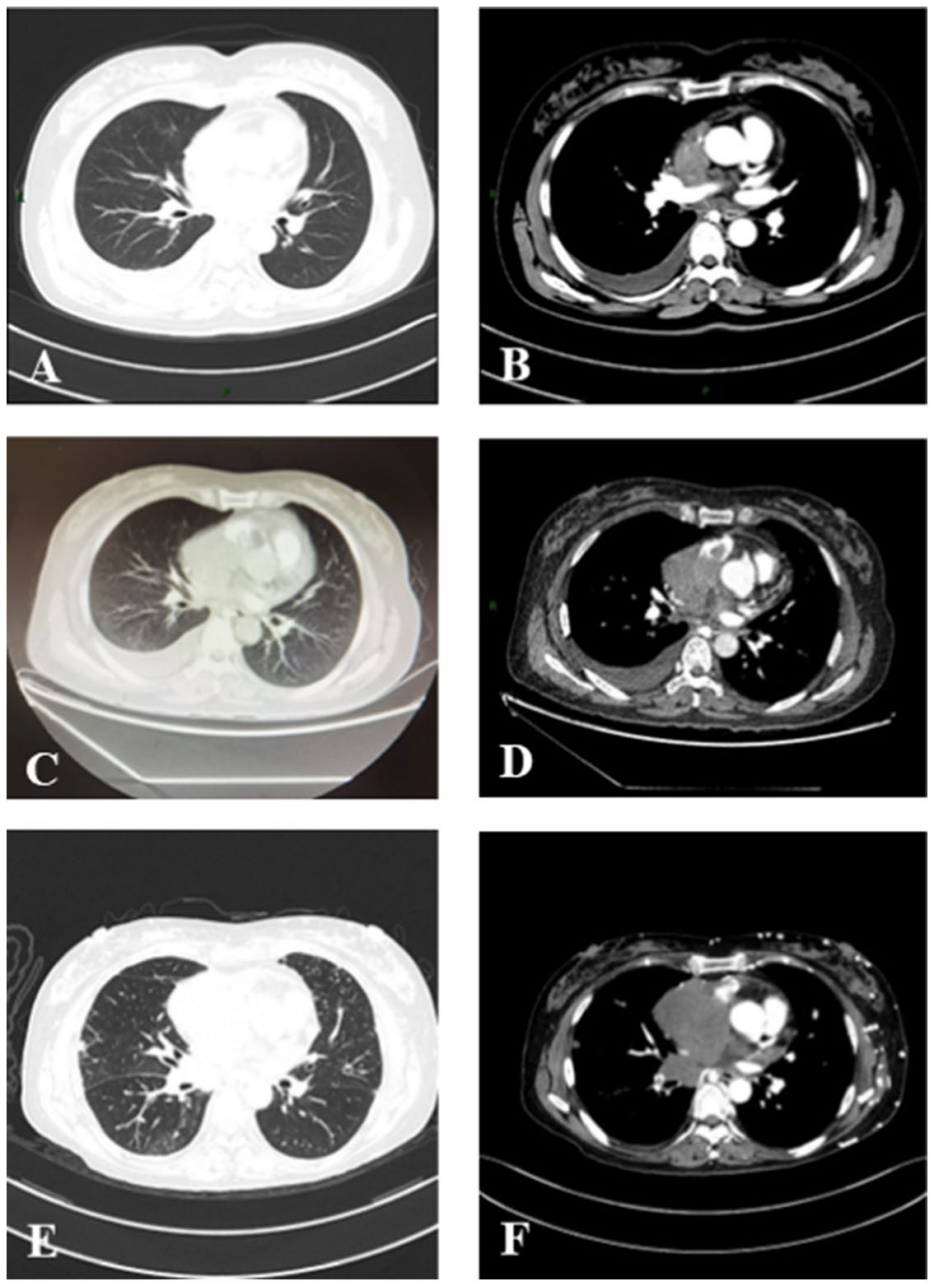
Serial chest CT scans of a patient with PCSS. **(A,B)** The initial CT scan on 14 December 2020 showed an irregular mass in the vena cava and right atrium with massive right pleural effusion. **(C,D)** A follow-up CT scan (15 January 2021) showed an enlarged mass measuring 4.0 × 3.4 × 7.6 cm in size in the vena cava and right atrium with pleural effusion on the right side. **(E,F)** CT scan on 8 March 2021 showed a rapidly enlarged mass in the vena cava and right atrium.

To further explore the etiology of pleural effusion, laboratory tests and thoracentesis were immediately performed. As shown in Table 1, the tuberculous infection T-cell spot test (T-SPOT) was positive. The serological tumor marker, neuron-specific enolase (NSE), was 17.2 ng/L (normal range: 0–16.3 ng/L). The D-D dimer was 2.16 mg/L (0–0.55 mg/L). The ANA (granule-type) titer clearly increased (1:1000). Pleural effusion was exudate with 90% monocyte classification. However, tubercle bacilli and tumor cells were not detected. Interestingly, the ANA (granule type) titer in pleural effusion was also elevated to 1:320. To further evaluate the cause of the neck thickness and edema in both upper limbs with an elevated D-D dimer, ultrasounds of venous, neck, and cardiovascular systems were arranged. No thrombosis was detected in the extremities. Transthoracic echocardiography revealed mild tricuspid valve regurgitation and strip echo in the right atrium, which was considered to be the Euclidean valve. During hospitalization, the patient suddenly experienced chest pain accompanied by dyspnea and decreased blood pressure (80/54 mmHg), without any apparent cause. Pulmonary thromboembolism (PTE) was highly suspected, and computed tomography pulmonary angiogram (CTPA) was immediately performed. The results revealed multiple filling defects in the right main and bilateral lower lobar pulmonary arteries. Furthermore, an irregular mass measuring 4.0 × 3.4 × 7.6 cm was discovered in the vena cava and the right atrium, with an invasion of the right main pulmonary artery (Figures 2A,B).

**Table 1:** Laboratory tests for etiology identification.

	Normal range	Result
Blood		
Total protein	63 ~ 82 g/L	69
LDH	313 ~ 618 U/L	487
D-D Dimer	0 ~ 0.55 mg/L	2.16
NSE	0 ~ 16.3 ng/mL	17.2
ANA	<1:100	1:1000
T-SPOT	A spot <6; B spot <6	A spot 45; B spot 62
Pleural effusion		
Number of nucleated cells	(0–8) × 10^6^/L	2,708 × 10^6^/L
Ratio of multinucleated cells	–	10%
Ratio of monocytes	–	90%
Total Protein	–	42 g/L
LDH	–	203 U/L
ADA	–	7.2 U/L
Smear of *mycobacterium tuberculosis*	Negative	Negative
GeneXpert MTB/RIF assay	Negative	Negative
Tubercle bacillus culture	Negative	Negative
ANA	Negative	1:320
Tumor cells	Negative	Negative
Lymph node biopsy		
Smear of *mycobacterium tuberculosis*	Negative	Negative
Tubercle bacillus culture	Negative	Negative
GeneXpert MTB/RIF assay	Negative	Negative
Smear of tumor cells	Negative	Negative

Based on these findings, the patient was transferred to the ICU and administered anticoagulation therapy with low molecular-weight heparin (LMWH). To identify the tumor type, thoracentesis and endobronchial ultrasound-guided transbronchial needle aspiration (EBUS-TBNA) were performed when the patient’s condition permitted. However, no positive results were obtained (Table 1). A multidisciplinary consultation still considered the possibility of a tumor, and the negative result might be attributed to the small size of the biopsy specimens.

**Figure 3:**
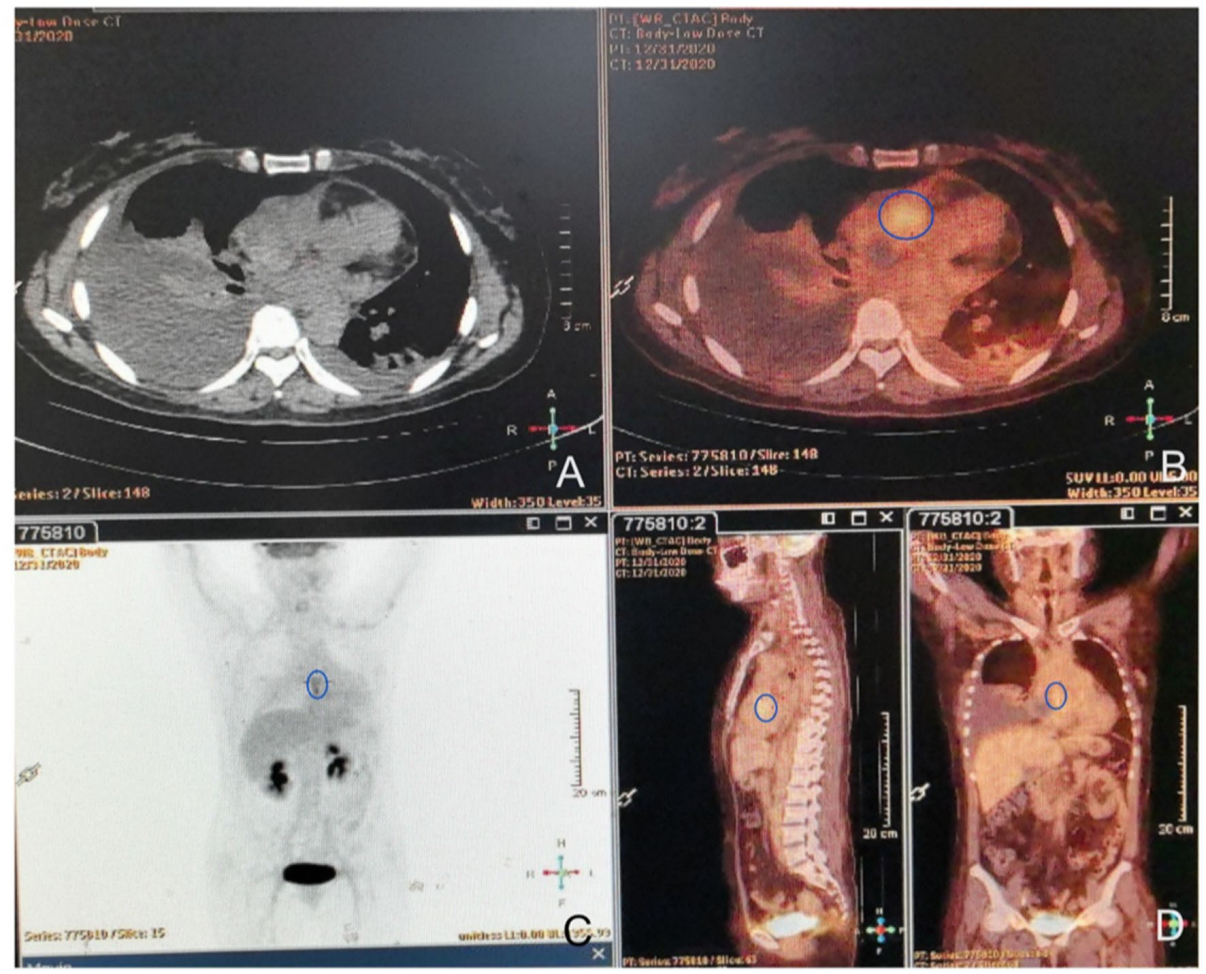
FDG-PET/CT images. **(A)** On the unenhanced CT, the mass was seen to invade the mediastinum and was contiguous with the pericardium. **(B)** Axial FDG/CT image. Mild uptake in the mass with an SUV_max_ of 4.2 SUV (circle area) was detected, and no extra-cardiac lesions were observed. **(C,D)** Whole-body maximum intensity projection (MIP) images show heterogeneous uptake (circle areas) in the expected location of the right heart.

**Figure 4:**
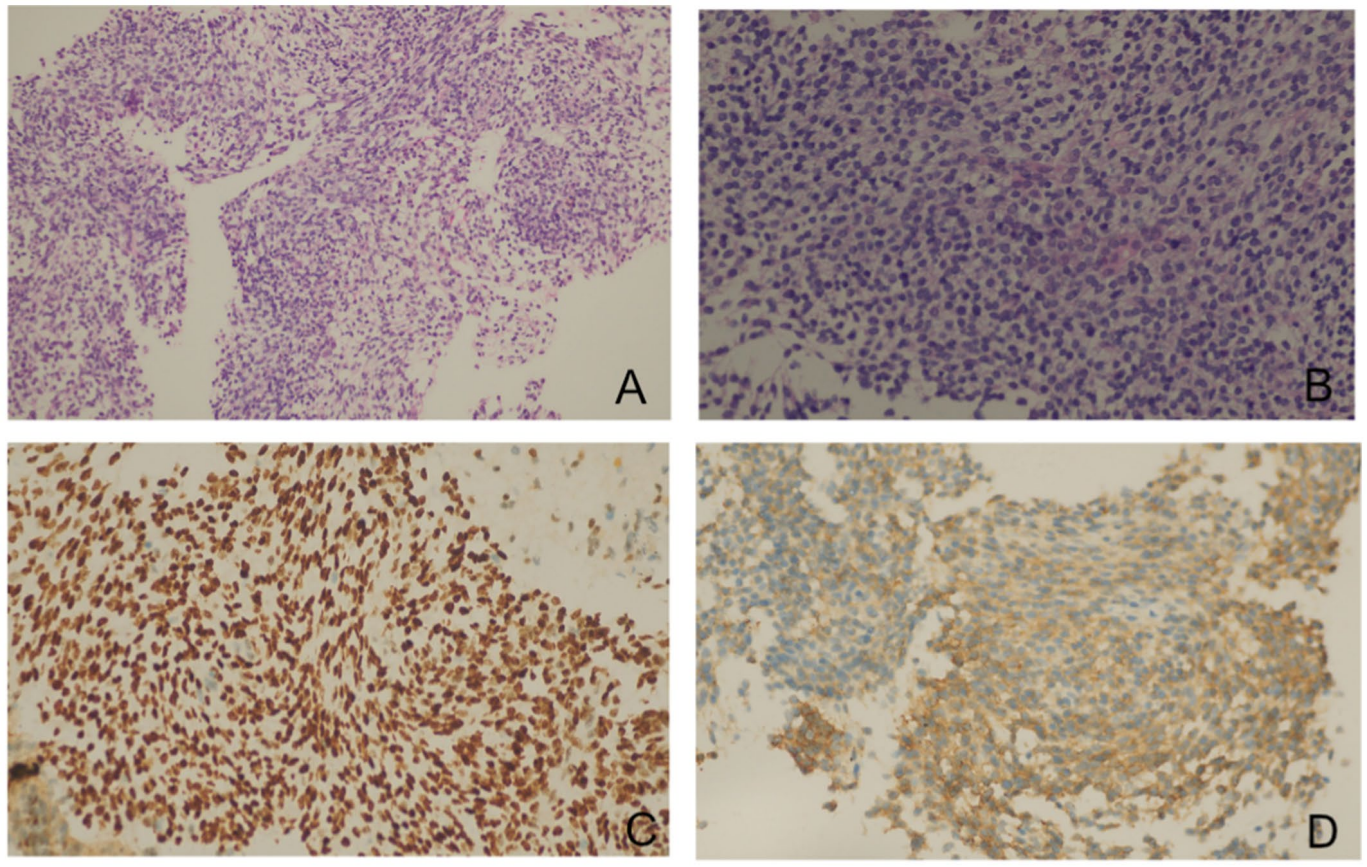
Pathology slides of the transbronchial specimens. Hematoxylin and eosin staining showed a biphasic tumor composed of spindle cells and epithelioid components [**(A)** × 10, **(B)** × 40]. Immunohistochemical examinations showed that the biphasic tumor cells were positive for TLE1 **(C)** and CD99 **(D)**.

As reported by Doğan C et al., high SUV_max_ values on PET/CT were associated with an increased diagnostic yield of transthoracic biopsy of lesions in the context of tumors (9). Meanwhile, EBUS-TBMB has been regarded as a safe and feasible technique for the biopsy of mediastinal or hilar lymphadenopathy or masses, with a superior overall diagnostic yield compared to EBUS-TBNA as a standalone technique (10). To obtain sufficient positive samples as accurately as possible, positron emission tomography/computed tomography (PET/CT) was conducted (Figure 3). After obtaining written informed consent, the patient was deeply sedated with a laryngeal mask airway. An Olympus EBUS bronchoscope (BF-UC180F, Olympus Medical Systems Corp., Tokyo, Japan) was used to assess the target mediastinal mass, and the higher metabolic heterogeneity was identified under the guidance of PET/CT (Figure 3). After identifying the target mass, the EBUS needle sheath was advanced through the bronchial wall into the lesion and moved forward and backward several times to enlarge the puncture hole. After extracting the EBUS needle and sheath, a closed mini-forceps (FB-433D, Olympus Medical Systems Corp., Tokyo, Japan) was advanced through the working channel of the EBUS bronchoscope to re-enter the mass through the pre-existing puncture hole. After penetrating the capsule of the lesion, the forceps were opened and advanced until reaching the target region. In the target region, the forceps were closed and pulled to obtain each specimen. This procedure was repeated 4–5 times under real-time imaging using EBUS. Finally, the closed forceps were withdrawn through the working channel. During this procedure, minor bleeding occurred; however, due to the continuous application of suction and ice saline washing, there was no active bleeding. Five tissue samples with an average dimension of 3–4 mm were obtained. Hematoxylin and eosin staining revealed variable areas of spindle cells and glandular-like epithelia (Figures 4A,B). Immunohistochemical examination showed that the biphasic tumor cells were positive for TLE-1 and CD99 (Figures 4C,D). Based on these findings, the patient was diagnosed with synovial sarcoma.

After the diagnosis, the patient was transferred to an oncology hospital for further treatment. During the follow-up visit, the patient underwent one cycle of systemic intravenous chemotherapy with a combination of doxorubicin and ifosfamide. However, the tumor progressed in the follow-up chest CT (Figure 2). Finally, the patient died in March 2021.

## Discussion

Synovial sarcoma (SS) is a rare malignant mesenchymal tumor with partial epithelial differentiation and is mostly located in the extremities (80–90%) (11). Although the main localizations are in the legs or arms, SS can appear in any part of the body, including the lungs, kidneys, cerebellum, parapharyngeal region, and heart (12). However, primary cardiac synovial sarcoma (PCSS) is extremely rare.

The diagnosis of PCSS is based on histopathological biopsy. Nevertheless, the specificity of the location makes biopsy extremely challenging. Therefore, it is particularly important to select non-invasive diagnostic or prognostic biomarkers. Here, we describe a case of suspected connective tissue disease (CTD) that was finally diagnosed as PCSS through EBUS-TBMB. In this case, the patient was highly suspected of having a CTD with elevated ANA titers in the blood (1:1000) and pleural effusion (1:320). When the treatment was planned, a sudden onset of dyspnea occurred, and CTPA revealed a mass in the vena cava and the right atrium with invasion of the right main pulmonary arteries. To the best of our knowledge, ANAs are a spectrum of autoantibodies that react with various nuclear and cytoplasmic components in normal human cells. They are very important markers for different autoimmune diseases such as systemic lupus erythematosus (SLE), scleroderma (SSc), polymyositis (PM), or mixed connective tissue disease (MCTD). However, some authors have proposed that ANAs are linked not only to autoimmune diseases but also to various types of cancer (13). Kaler et al. (14) also reported the value of ANAs in the early diagnosis and prognosis prediction of carcinoma. To date, there have been no reports on the value of ANAs in the diagnosis and prognosis of patients with PCSS. The present study identified significantly elevated titers of ANAs in patients with confirmed PCSS, whereas no evidence supporting the presence of autoimmune disorders was found. Unfortunately, the patient was not followed up for changes in ANAs titers during subsequent hospitalization and treatment, and there was no direct evidence to consolidate the correlation between ANAs and PCSS. Therefore, future studies should focus on the relationship between autoimmune markers and tumor diagnosis or prognosis, especially for PCSS.

Traditionally, open incisional biopsy has been performed in some cases of PCSS (7, 8). However, to the best of our knowledge, this procedure requires general anesthesia and may lead to more complications such as hematoma and wound infection. It may also cause tumor spread and delay neoadjuvant treatment (15). Less invasive soft tissue tumor biopsy methods should be explored. EBUS-TBNA has been developed to be an integral tool for the diagnosis and staging of lung cancer and other diseases involving mediastinal lymphadenopathy. However, because of the small sample size and the providers’ skills, the sensitivity varied from 33.3 to 64.5% (16). Compared to EBUS-TBNA, EBUS-TBMB has been verified as a more feasible and safe technique for enlarged mediastinal/hilar lymphadenopathy or masses (17, 18). PET/CT-guided biopsies have been reported to help in difficult situations, especially when it is important to know which part of the tumor is active or which lesion is active in patients with multiple widespread lesions (19). PET/CT guidance can maximize the diagnostic yield of image-guided procedures and direct needle insertion into the viable area of the lesion (20). In our case, we obtained a negative result with EBUS-TBNA. To improve the chances of obtaining positive specimens, we performed modified EBUS-TBMB with PET/CT guidance, which can provide more precise samples with greater volume.

Due to its dual epithelial and mesenchymal nature, immunohistochemistry is necessary to make a definite diagnosis of SS. A recent systematic review examining the role of TLE1 as a diagnostic biomarker for SS found that the mean sensitivity and specificity of TLE1 in detecting SS were 94 and 81%, respectively (3, 21). CD99 has also been detected in 60% of SS cases (4). In our case, the patient was diagnosed with SS based on specific histopathological features and positive expression of TLE1 and CD99 in immunohistochemistry.

Due to its low prevalence, no prospective cohort or randomized controlled trial is feasible to evaluate the survival outcomes of orphan disease. A median survival of 7 months was identified based on the SEER database with a population of 442 (22). Surgical resection with neoadjuvant chemotherapy is the primary treatment option (23, 24). However, because of the infiltrative nature and extent of the neoplasm, it is difficult to perform the complete resection of the cardiac tumor. First-line systemic chemotherapy was administered with adriamycin at a dose of 75 mg/m^2^ for 3 days and ifosfamide at a dose of 10 g/m^2^ divided over 4 to 5 days. The second-line regimen consists of a combination of gemcitabine and docetaxel (25). Doxorubicin was thought to be a cornerstone treatment for advanced soft tissue sarcoma (26). Meanwhile, new therapeutic strategies such as immunotherapy and targeted therapy with pazopanib and trabectedin have been proven to improve the prognosis (25, 27). Moreover, it is controversial whether patients with extensive infiltration of CSS should have a heart transplant if a suitable heart donor is available (28). In our case, due to infiltration of the superior vena cava and cervical lymph nodes, the patient had no opportunity for surgical resection. Systemic intravenous chemotherapy with a combination of doxorubicin and ifosfamide was administered. Unfortunately, the patient did not respond to this therapy and died 3 months later. More effective therapies with less toxicity need to be explored for poor prognosis.

This study had some limitations. The rarity of the PCSS limits the generalizability of the findings, and the case report format inherently restricts its broader applicability. The advanced stage at which the patient was diagnosed with reduced treatment options could skew the perceptions of the disease’s management and outcomes. Additionally, the lack of a comprehensive autoimmune assessment leaves a gap in the understanding of the relationship between CTD and malignancy, which could have implications for patient management and prognosis. Further exploration is essential to understand the relationship between autoimmune markers and tumor diagnosis, which could provide insights into early detection and personalized treatment strategies.

## Conclusion

In summary, we report a case of PCSS in a patient presenting with a mass in the vena cava and the right atrium, accompanied by pleural effusion and elevated ANA titers. Clinicians need to refine their understanding of the characteristics of PCSS, especially in patients with pleural effusion and elevated ANA titers. When other evidence of CTD is lacking, malignant tumors should be considered, and histopathological biopsy should be performed. For PCSS, the application of EBUS-TBMB, especially with the guidance of PET/CT, represents a novel diagnostic approach that may improve the accuracy in complex cases. Early diagnosis and surgical resection are critical for the prognosis of PCSS. However, owing to a lack of sufficient awareness of the disease, most PCSS cases are diagnosed at an advanced stage. This case offers a clear description that can assist clinicians in identifying similar cases in their clinical practice.

## Data Availability

The original contributions presented in the study are included in the article/supplementary material, further inquiries can be directed to the corresponding author/s.
